# Builder, Expert, Disruptor, Leader: The Many Roles of People with Lived Experience

**DOI:** 10.5334/ijic.7696

**Published:** 2023-08-23

**Authors:** Robin Miller, Nieves Ehrenberg, Caroline Jackson, Heather Penwarden, Viktoria Stein, Wilma van der Vlegel-Brouwer, Anne Wojtak

**Affiliations:** 1 University of Birmingham, United Kingdom; 2 VM Partners Integrating Health and Care, Portugal; 3 Dementia Friendly Honiton & Public Governor Royal Devon NHS Foundation Trust, United Kingdom; 4 Leiden University Medical Centre, Netherlands; 5 SevenSenses Institute, Netherlands; 6 University of Toronto, Canada

**Keywords:** co-production, lived experience, citizen leader

## Abstract

People with lived experience of health and social care, including family carers, should be at the heart of integrated care policy and practice. One of the challenges to achieving such co-production is insufficient clarity and limited understanding of the different roles that people with lived experience are asked or choose to undertake. Following research and workshops, four roles have been identified – community builder, improvement expert, disruptor/advocate, and citizen leader. Recognising the distinct contribution and demands of these roles will enable appropriate support and development for people with lived experience and the professionals and managers with whom they collaborate.

## Introduction

*“One of the things in my newly found confidence is to question how the organisations that I work for are developing future leaders in my space. This includes the growth and development of the more vulnerable people that aren’t being reached at the moment and how are we lifting them up to be able to enjoy the confidence that I have now.” (Person with lived experience)*

The active involvement of people who have experience of health and social care, including family carers (hereafter collectively referred to as *people with lived experience*) has been rightly adopted as a fundamental principle of integrated care [1,2]. Despite its centrality, it is rare though for policy makers and programme managers to truly demonstrate co-production in how they design and implement integrated care [3]. Co-production requires people with lived experience to be at the planning table from the outset so that they can shape not only *how* questions should be answered, but *what* the questions should be [4]. Such limitations at the strategic level mirror those challenges which many people experience in their own care, with professionals and teams finding it difficult to reframe their thinking and practice to put people truly at the centre of decision making [5]. Similar challenges have also been traditionally found in research, although there is increasing commitment to engaging people with lived experience in designing, undertaking and interpreting research and not solely being the ‘subject’ [6].

Obstacles to co-production are many and long-standing. Frequently these are practical – poor communication, inaccessible venues, insufficient capacity, digital exclusion, and limited financial support to meet costs such as travel, alternative care arrangements, and digital access. Others, and arguably these are the most problematic, relate to cultural norms within professional and managerial practice which view expertise based on lived experience as being of lesser importance to that based on formal education and management status. These obstacles, and the potential solutions to addressing them, were recently studied in an international research project (7). The idea arose through discussions with a community advisory board of people with lived experience, and how their leadership differed (or not) from leadership demonstrated by professionals and managers. The project heard the stories of people with lived experience from many countries and highlighted not only the many challenges and barriers but also good examples of how co-production can work in practice [7].

A key issue which emerged in the research was how people described the roles they undertook when seeking to influence integrated care. It is important to note that some participants did not consider the title of such roles important as such. What mattered instead was the impact of their efforts and how this improved future care for people and communities. For others, considering alternative titles for their roles provided an opportunity to reflect on how they sought to influence and on how others might understand their contribution. To further explore these issues, the research team facilitated a workshop at ICIC 2023 which was attended by an enthusiastic group of people with lived experience, practitioners, and researchers.

A World Café approach was used to enable workshop participants to explore the roles of Disruptor/Advocate; Community Builder; Improvement Expert; and, Citizen Leader, all of which had been identified through the stories of research participants.

## Community Builder

Community builders seek to find and connect people with similar interests and challenges to provide mutual support and shared representation. This could for example be related to living with the same condition, being subjected to common discrimination and social exclusion, and/or being a member of a faith group or indigenous community. The role requires an understanding of what motivates people to seek collective support and camaraderie, how group dynamics (and tensions) work in practice, and how to encourage people to develop open and trusting relationships. A risk for community builders is that they are seen by others as taking the sole responsibility for maintaining networks and solving all problems – the ability to engage others and encourage (in some cases challenge) them to recognise their own agency and assets was therefore an important skill.

## Improvement expert

A common access point for people with lived experience is being invited to contribute to a discrete quality initiative, such as reviewing an integrated care pathway or improving the physical environment of a service. This invite is based on having the condition(s) or experience(s) in question, receiving related treatment or support, and in some cases providing positive or negative feedback regarding their care to professionals. People will often be invited to participate once the focus has been identified and improvement processes are underway, therefore limiting their ability to influence. There are though examples in which people with lived experience are invited to define the problem and scope out what should be considered – this gives greater opportunity for co-production. Core skills for an improvement expert include articulating relevant experiences to other members of the process, being confident in challenging the perspectives of professionals, and understanding common approaches to analysis and sharing data.

## Disruptor/advocate

Despite centrality of co-production to integrated care, there are still many instances in which people’s views are not heard or acted on by those with power to change policy and practice. As a result, people with lived experience can be forced to adopt more radical strategies to ensure their voices are heard. These include working with the media to increase awareness of current failings, making legal challenges to the status quo, and lobbying politicians to introduce new mandates and invest resources. Assertiveness, persistence, communication, and the ability to engage and represent the perspectives of others are central to such disruptive activity. A dilemma for disruptor/advocates is how to balance the need to confront those with power whilst maintaining opportunity for future collaboration and constructive dialogue. Being a voice outside the established processes can feel a lonely endeavour, particularly as change may take a considerable time, and developing peer networks with which to share challenges and frustrations are therefore vital.

## Citizen leader

Citizen leadership was the most divisive of the roles discussed. Many people with lived experience do not see themselves as leaders as they connect leadership with a status that they do not recognise in their own lives. Similarly, some see their contribution as being based on their experience and identity as a patient, a carer, or a survivor rather than as a citizen per se. For others though, whilst they would not commonly describe themselves as leaders, do see that the processes of leadership (i.e., sharing a vision, encouraging others to engage, and facilitating diverse contributions) reflects what they do. Still other people with lived experience have already taken the identity of leaders in their work and in some cases have formal roles using this title. The skills of citizen leaders were shared with other roles but with particular emphasis on the ability to use stories to encourage people and professionals to connect intellectually and emotionally, actively reach out to those who feel most disenfranchised, and to listen and represent the stories of others.

## Turning roles into reality

The complexity of these roles highlights the sophisticated set of skills and underpinning knowledge required by people with lived experience. There are examples in which services have clearly outlined their expectations (particularly around quality improvement) and provided corresponding induction, training and/or peer mentoring to help people to grow in confidence and skills. However, mostly people with lived experience have to work out for themselves what is required and seek their own opportunities for development. There is therefore a need for defined support and training which is accessible and resourced, and which relates to the roles outlined above (and others which may emerge). Development should not, however, be limited to those with lived experience. The need is just as great for those who fund, manage, and deliver health and social care systems. Time and time again, people with lived experience report tokenism in the extent to which they can shape integrated care policy and practice. This leads to frustration and despondency that the espoused aspiration to change is not authentic. Whilst some people with lived experience take the difficult route of advocating and disrupting, others will withdraw from trying to improve integrated care altogether.

True co-production requires services and governments to commit to sustained engagement, a willingness to listen to different and at times conflicting voices, and a humility to recognise when their established wisdom has been wrong. Whilst this undoubtedly entails individual and collective challenges, these are outweighed by the opportunities to improve outcomes, to address inequalities, and for professionals and managers to gain greater satisfaction from their work. Better understanding the breadth of roles that people with lived experience undertake, investing in their capacity and skills, and supporting managers and professionals to be receptive to their contribution will be of benefit to all.
